# Potential Uses of Cord Blood in Cardiac Surgery

**DOI:** 10.1155/2012/568132

**Published:** 2012-02-16

**Authors:** Ralph S. Mosca

**Affiliations:** Department of Cardiothoracic Surgery, Pediatric, and Adult Congenital Cardiac Surgery, NYU Langone Medical Center, 530 First Avenue, Suite 9V, New York, NY 10016, USA

## Abstract

Despite advances in the fields of prevention, medical intervention and surgical therapy, cardiovascular disease remains a major public healthcare issue. A promising area of research is the potential application of regenerative therapies with pluripotential stem cells to reduce the burden of heart disease and its sequelae. Umbilical cord blood, a rich source of multiple populations of nonembryonic stem cells, will be a valuable resource and has the potential to advance therapeutic options for patients with acquired and congenital heart disease.

## 1. Introduction

Atherosclerotic heart disease and chronic heart failure remain a major worldwide health problem [1]. According to the American Heart Association statistics, over one in three American adults have one or more types of cardiovascular disease resulting in an average of 1 death every 39 seconds. In addition, millions of people are now living with the results of palliated congenital heart disease such as artificial valves and conduits and declining ventricular function [2]. Significant advances in medical care; pharmacologic therapy, interventional catheterization, and surgical procedures; coronary artery bypass grafting and hybrid techniques, ventricular restoration, ventricular assist devices, and organ transplantation have improved the lives of many. Yet these therapies are expensive, yield variable results and are not available or applicable to all clinical states. Finally, many of these modalities do not get to the “heart” of the matter: a reduction in the effective contractile architecture or the need for true autologous cardiac structures.

## 2. Umbilical Cord Blood Stem Cells

 Undifferentiated cells capable of self-renewal and developing into various tissues and organs are referred to as stem cells (SC) [3]. Multiple sources of human stem cells, embryonic and nonembryonic (bone marrow, adipose tissue and umbilical cord blood), have been characterized and studied in preclinical scenarios. Each has its own particular advantageous and disadvantageous properties. Embryonic stem cells, for example, have great potential for regeneration given their high content of pluripotent cells. Currently mired in political and ethical debate, their use is further complicated by the risk of teratoma formation [4]. Stem cells from adult sources (bone and adipose tissue) can provide an alternative but may carry a higher risk of somatic mutations [5, 6]. Umbilical cord blood contains various populations of stem cells and progenitors including hematopoietic stem cells (HSCs), mesenchymal stem cells (MSCs), endothelial colony forming cells (ECFCs) and more primitive unrestricted somatic stem cells (USCCs) [7, 8]. Given its more naïve state and “younger” environmental exposure UCB retains great pluripotentiality, less immunogenicity, and fewer mutations. Conversely, it may be more difficult to harvest under sterile conditions and in sufficient volume. Finally, although UCB is a rich source of many populations of primitive stem cells, the yield of each cell line from any given sample may vary considerably.

## 3. Transfusion

Despite the increasing attempts to limit the use of exogenous blood and its components, allogeneic transfusion remains a necessity in a significant percentage of hospitalized patients. This is particularly true when caring for patients at the extremes of ages with cardiovascular disease. Umbilical cord blood (UCB) is rich in adult and fetal hemoglobin cells as well as platelets and other cellular constituents and offers minimal immunologic reaction [9]. Given fetal hemoglobin's enhanced oxygen affinity UCB offers an ideal blood substitute and has been used to treat adults suffering from anemia associated with diabetes mellitus, rheumatoid arthritis, and infectious diseases such as malaria, leprosy, tuberculosis and HIV. Thalassemia and sickle cell patients have also benefited from the stem cells derived from UCB [10]. Major limitations to its widespread use include, the relatively small volume of blood obtained (approximately 150 cc's) from one placenta, shelf life (approximately 30 days), consent for and administration of serologic tests from the pregnant woman prior to delivery, issues of sterility (vaginal delivery > caesarian section), and increased cost (6x's the expense of voluntarily donated allogeneic RBC) during collection and cultural and superstitious barriers [11, 12].

 Premature infants are often born with multiple major medical problems including cardiopulmonary disease requiring long-term hospitalization, interventional procedures, and blood transfusion. In the past, although the collection and preparation of placental and umbilical cord blood was technically possible, a number of questions were raised regarding its safety and utility (given the variable rates of eventual transfusions) [11]. The probability of needing and benefiting from UCB transfusion may now be possible to estimate from parameters such as gestational age, birth weight, and Apgar scores [13].

Many congenital defects requiring postnatal surgical correction are now diagnosed antenatally. A significant number of these babies will require blood transfusion. A recent study investigating the use of UCB in neonates undergoing general surgical procedures found this technique useful and noted that 64% of patients avoided allotransfusion [14]. In addition, reports are now appearing of neonates undergoing the repair of complicated congenital cardiac defects using AUCB to prime the extracorporeal circuit [15].

## 4. Acquired Heart Disease

Ischemic heart disease and chronic congestive heart failure are a major worldwide healthcare problem [16]. Myocardial damage produces a loss of myocytes and can result in progressive chamber thinning and dilation. Medical and surgical therapies attempt to reperfuse potentially viable myocardium, pharmacologically or mechanically augment cardiac output or replace the heart. None of these treatments are aimed at regenerating the myocyte and other structural cardiac components.

On the heels of major successes in allogeneic stem cell transplantation (AlSCT) and more recently related and unrelated umbilical cord blood transplantation (UCBT) for numerous malignant and nonmalignant diseases, many researchers and clinicians see great hope for application in regenerative therapy [17].

An animal model provided the initial evidence that cardiac structure and contractility could be improved with cell transplantation. Fetal cardiomyocytes transplanted into rat hearts following cryoinjury improved systolic function and limited scar progression [18]. Follow-up studies reported similar results in animal models after transplantation of cardiomyocytes, skeletal myoblasts, smooth muscle cells, and c-kit-positive bone marrow stem cells [19, 20]. It appeared that replacement of the functional myocyte unit in injured myocardium would become a clinical reality.

Early human studies demonstrated the feasibility of cell transplantation (skeletal myoblasts) into patients with ischemic heart failure [21]. Outcomes in these small studies were variable, and risks seemed to be centered around and increased incidence of postoperative arrhythmias [22]. The multicenter MAGIC (myoblast autologous grafting in ischemic cardiomyopathy) trial was then undertaken in Europe [23]. Inclusion criteria were severe left ventricular dysfunction, postinfarction scar, and concomitant coronary artery bypass grafting (CABG). Thigh myoblasts were injected into and on the borders of the scar following bypass grafting. There was no improvement, at the 6-month study point, in left ventricular (LV) function; however, a significant decrease in LV end-diastolic and systolic volumes were observed. Highly variable results were also obtained when investigators utilized bone marrow-derived cells during CABG [24, 25].

A reexamination of the animal and human data showed that only a small percentage (1%) of transplanted cells remain engrafted at the original site a few weeks following administration [26]. Cell loses occur due to wash out during application and death of cells initially residing in this ischemic environment. Thus, the original hope of repopulating injured areas of myocardium with myocytes recreating functional contractile units has yet to be realized. Despite this disappointment beneficial effects on cardiac remodeling have been consistently observed. Currently, studies are underway to define possible paracrine effects (inducing angiogenesis, restoring and stabilizing the extracellular matrix, and limiting apoptosis) caused by the small mass of surviving cells that may limit maladaptive cardiac remodeling [27–29].

## 5. Congenital Heart Disease

Heart defects are the most common congenital affliction and occur in approximately 14 out of 1000 live births [1, 30]. The various structural lesions can result in profound physiologic perturbations as a result of pulmonary over circulation, cyanosis, and pressure or volume overload of the cardiac chambers. As a consequence, congenital heart disease remains the number one cause of death in the first year of life [31]. Approximately 25,000 surgical procedures are performed each year in an attempt to correct or palliate these conditions. Children with congenital heart defects often require a series of operations in the first few years of life.

Chronic pressure or volume overload lesions of either the right or left side of the heart; aortic and pulmonary valve disease, coarctation of the aorta, single ventricle lesions may produce significant myocardial dysfunction resulting in chronic congestive failure. Often requiring multiple interventions, they are candidates for cell and tissue therapy to help restore contractile function.

Multiple conditions may be associated with underdevelopment or absence of the semilunar valves. Currently, valve repair is attempted but often culminates in replacement of the aortic or pulmonary valves with bioprosthetic or mechanical prostheses (valves and or valved conduits). Although hemodynamically adequate, these valves often fail by calcification and or tissue ingrowth relatively rapidly and have no capacity for growth thus necessitating multiple reoperations, and many require systemic anticoagulation with its attendant risks. A great deal of research is now underway to develop tissue-engineered valves seeded with a patient's own cells with the potential for growth and lack of immunogenicity. Combining the techniques of cell harvesting, cell augmentation, and tissue-engineering the hope is to bank suitable stem cells and “grow” them into useable valve substitutes. Scaffolds made of decellularized xenografts or tissue engineered biodegradable matrices have been designed to replace traditional valve supports [32]. Autologous or donated umbilical cord blood could serve as an ideal source of donor cells.

Patients born with univentricular hearts (tricuspid and or pulmonary atresia as well as hypoplastic left heart syndrome) require multiple operative procedures utilizing prosthetic shunts culminating in a Fontan procedure. Often a circumferential prosthetic tube is placed to channel the deoxygenated blood from the abdomen and lower extremities directly into the lungs, thereby bypassing the missing pulmonary ventricle. Similarly, patches or baffles of artificial material (Dacron, PTFE) are used on a daily basis in pediatric cardiac surgery to connect or reroute blood flow within the heart. These conduits and patches have no growth potential, carry a risk of thrombosis as well as infection, and may require systemic anticoagulation [33]. Tissue-engineered vascular grafts, seeded with autologous endothelial stem cells, could produce the optimal vascular graft with the ability to keep pace with somatic growth, eliminate the need for reoperation, and greatly reduce the risk of postoperative thrombosis and infection. Following successful animal studies, tissue-engineered vascular grafts (TEVGs) were implanted in humans undergoing total cavopulmonary connections demonstrating the feasibility of this technique [34].

The number of adults living with congenital heart disease now outnumbers children afflicted with these conditions [35]. Many of these patients were operated upon in an earlier era when medical and surgical techniques were not as well refined. For example, repairs of Tetralogy of Fallot were routinely performed at an older age through large right ventricular incisions and employing long transannular patches with long cardiopulmonary bypass times and suboptimal myocardial protection. As a result, they often exhibit severe right ventricular dilation and fibrosis. In addition as this patient population ages and their comorbidities increase (obesity, diabetes mellitus, etc.) they may develop left ventricular dysfunction as well as a consequence of epicardial coronary artery disease. Given the existing shortage of hearts for cardiac replacement and the uncertainties of ventricular device support in this population, restoration of their ventricular function with cell-based therapies [36] could have significant impact on the lives of many.

## 6. Outlook

Umbilical cord blood is a relatively easily obtainable source of multiple populations of pluripotent cells seemingly less hampered by mutations and immunogenicity. When harvested it provides a readily available, lifelong source of cells for therapy. It is rapidly becoming the standard of care for use in hematologic and oncologic disorders. Although the volume and numbers of particular cells may be limited in any one sample, transplantation of multiple units from different donors may be required and has been accomplished with good results [37–39], and recent strides in stem cell expansion [40] and transduction [41] should augment supplies.

Cardiovascular disease remains one of the world's largest and growing healthcare issues. The fields of cell transplantation, tissue engineering, and regenerative medicine are poised to impact heart disease in a similar way to that seen in oncohematologic disorders and inborn errors of metabolism. Despite the genetic, biological, and engineering complexities involved, restoration of ventricular function and the development of living and growing vascular grafts and valves is becoming a reality. Umbilical cord blood will certainly play a central role in “seeding” these accomplishments.
